# Intranasal human-recombinant NGF administration improves outcome in children with post-traumatic unresponsive wakefulness syndrome

**DOI:** 10.1186/s13062-023-00418-1

**Published:** 2023-10-03

**Authors:** Antonio Gatto, Lavinia Capossela, Giorgio Conti, Gemma Eftimiadi, Serena Ferretti, Luigi Manni, Antonietta Curatola, Benedetta Graglia, Lorenzo Di Sarno, Maria Lucia Calcagni, Daniela Di Giuda, Stefano Cecere, Domenico Marco Romeo, Marzia Soligo, Enzo Picconi, Marco Piastra, Giacomo Della Marca, Susanna Staccioli, Antonio Ruggiero, Fabrizio Cocciolillo, Silvia Pulitanò, Antonio Chiaretti

**Affiliations:** 1 Dipartimento di Pediatria, Fondazione Policlinico Universitario “A. Gemelli” IRCCS, Rome, Italy; 2 Dipartimento di Pediatria, Università Cattolica del Sacro Cuore, Rome, Italy; 3 Terapia Intensiva Pediatrica, Dipartimento di Scienze dell’Emergenza, Anestesiologiche e Rianimazione, Fondazione Policlinico Universitario “A. Gemelli” IRCCS, Rome, Italy; 4 Istituto di Farmacologia Traslazionale, Consiglio Nazionale delle Ricerche (CNR), Rome, Italy; 5 UOC di Medicina Nucleare, Fondazione Policlinico Universitario “A. Gemelli” IRCCS - Università Cattolica del Sacro Cuore, Rome, Italy; 6 Unità di Neurologia Pediatrica, Fondazione Policlinico Universitario “A. Gemelli” IRCCS, Rome, Italy; 7 Dipartimento di Scienze dell’Invecchiamento, Neurologiche, Ortopediche e della Testa-Collo, Fondazione Policlinico Universitario Agostino Gemelli, IRCCS, Rome, Italy; 8 Dipartimento di Neuroriabilitazione Intensiva, Ospedale Pediatrico “Bambino Gesù”, Rome, Italy; 9 Oncologia Pediatrica, Fondazione Policlinico Universitario A.Gemelli IRCCS - Dipartimento Scienze della Salute della Donna, del Bambino e di Sanità Pubblica, Università Cattolica del Sacro Cuore, Rome, Italy; 10 Department of Women’s Health Sciences, Fondazione Policlinico Universitario A. Gemelli – IRCCS, Largo Agostino Gemelli 8, 00168 Rome, Italy

**Keywords:** Human-recombinant nerve growth factor, Traumatic brain injury, Unresponsive wakefulness syndrome, Intranasal administration

## Abstract

**Background:**

Severe traumatic brain injury (TBI) is one of the most dramatic events in pediatric age and, despite advanced neuro-intensive care, the survival rate of these patients remains low. Children suffering from severe TBI show long-term sequelae, more pronounced in behavioral, neurological and neuropsychological functions leading to, in the most severe cases, an unresponsive wakefulness syndrome (UWS). Currently, no effective treatments can restore neuronal loss or produce significant improvement in these patients. In experimental animal models, human- recombinant Nerve Growth Factor (hr-NGF) promotes neural recovery supporting neuronal growth, differentiation and survival of brain cells and up-regulating the neurogenesis-associated processes. Only a few studies reported the efficacy of intranasal hr-NGF administration in children with post- traumatic UWS.

**Methods:**

Children with the diagnosis of post-traumatic UWS were enrolled. These patients underwent a treatment with intranasal hr-NGF administration, at a total dose of 50 gamma/kg, three times a day for 7 consecutive days. The treatment schedule was performed for 4 cycles, at one month distance each. Neuroradiogical evaluation by Positron Emission Tomography scan (PET), Single Photon Emission Computed Tomography (SPECT), Electroencephalography (EEG), and Power Spectral Density (PSD) was determined before the treatment and one month after the end. Neurological assessment was also deepened by using modified Ashworth Scale, Gross Motor Function Measure, and Disability Rating Scale.

**Results:**

Three children with post-traumatic UWS were treated. hr-NGF administration improved functional (PET and SPECT) and electrophysiological (EEG and PSD) assessment. Also clinical conditions improved, mainly for the reduction of spasticity and with the acquisition of voluntary movements, facial mimicry, attention and verbal comprehension, ability to cry, cough reflex, oral motility, and feeding capacity, with a significant improvement of their neurological scores. No side effects were reported.

**Conclusion:**

These promising results and the ease of administration of this treatment make it worthwhile to be investigated further, mainly in the early stages from severe TBI and in patients with better baseline neurological conditions, to explore more thoroughly the benefits of this new approach on neuronal function recovery after traumatic brain damage.

**Supplementary Information:**

The online version contains supplementary material available at 10.1186/s13062-023-00418-1.

## Introduction

Traumatic brain injury (TBI) is the main cause of disability and mortality among children and young adults [1]. Each year more than 55 million people, worldwide, are estimated to experience a mild TBI and 5.5 million a severe TBI [2]. The most recent data from the CDC, published in 2019, estimates 2.87 million emergency department visits, hospitalizations, and deaths in the United States, annually, related to TBI [3]. Data suggest that some groups of people are at greater risk of death from a TBI or experiencing long-term neurological problems after injury [4]. Rates of TBI per 100,000 individuals is highest for elderly people (> 75 years old), very young children (0 to 4 years), and teens/young adults (aged 15 to 24 years). The pathophysiology of TBI is quite complex and lead to a significant damage of both gray and white matter. The mechanical insult produces primary injuries on neurons (failure ion homeostasis control, cell depolarization and excitotoxicity), endothelial cells (blood vessel damage), astrocytes and glia (release of proinflammatory cytokine and chemokines), mitochondrial function (energy failure, calcium accumulation and release of oxygen species), oligodendrocytes (demyelination) and axons (axonal degeneration, protein aggregation). This primary event led to an excessive acute inflammation, a reduction of the cerebral blood flow resulting in hypoxia, hypoglycemia, and breakdown of the blood–brain barrier [5]. Secondary brain injury is mainly related to reperfusion, hypoxia, neuroinflammation, oxidative damage oedema and ischaemia [5, 6]. Secondary brain damage frequently causes the loss of cerebral neurons and can be associated with a progressive decrease in cognitive and motor functions. Children suffering from severe TBI show long-term sequelae, more pronounced in behavioral, neurological and neuropsychological functions leading to, in the most severe cases, an unresponsive wakefulness syndrome (UWS) [7]. Despite of advances in technology and increasing knowledge about pathophysiology of TBI, in the clinical practice there are no effective therapies to restore neuronal damage or produce significant clinical improvement after brain injury. In the last decades, researchers are engaged in looking for effective therapeutic strategies to improve neurologic outcome in these patients. However, therapies that have been demonstrated to improve the outcomes after TBI are still limited, especially in the management of severe TBI. New treatments with good therapeutic windows and multiple mechanism of action able to counteract early and chronic pathophysiological events are urgently needed [5, 8].

It is known that after TBI neuroplasticity mechanisms and brain remodeling processes are spontaneously activated in order to increase functional recovery of the brain. Among these mechanisms, the secretion of neuropeptides and their distribution to the damaged cerebral area is one of the main events that enhance neurogenesis and cognitive functions [9, 10]*.* The role of these neuropeptides has been investigated and among them NGF has gained significant attention as potential new treatment for TBI. It has been demonstrated that NGF has the potential to act on the primary and secondary events that are part of the acute and chronic clinical manifestation of TBI [11]. Several studies have supported the use of this neurotrophin in TBI on the basis of the following pharmacological evidences: (a) its effect on cells and targets that are involved in the primary and secondary injuries. This led to a significant reduction at cellular and molecular level of neuroinflammation, protein aggregation, mitochondrial dysfunctions and an increase of angiogenesis and oligodendrocyte protection, as recently reported in literature [12]; (b) evidence that demonstrate the release and the neuroprotective action of endogenous NGF after TBI in in vivo model and in patients [13–18]; (c) efficacy in in vivo model of TBI following intraventricular and intranasal administration showing the significant neuroprotective effect, the ability to reduce cerebral edema, reducing inflammatory cascade, protein aggregation and deposition [19–22]. Only the studies conducted by Young et al. reported a negative outcome of NGF action [23]. More recently our group demonstrated a promising pharmacological activity of acute intranasal NGF in the prevention of TBI motor dysfunction and neuroinflammation in young rats. These results suggested a potential use as acute treatment immediately after traumatic injuries in order to block the primary and secondary damage of TBI [24]; **d)** preliminary clinical data demonstrated that intraventricular and intranasal NGF administration has been safe and effective in improving clinical functions in children with severe long-term neurological sequelae after TBI and meningitis, respectively [12, 25–28]. Recently, promising results have also been reported by the combined treatment of intranasal human-recombinant NGF (hr-NGF) and transcranial direct current stimulation (tDCS) in 3 children with chronic vegetative state after out-of-hospital cardiac arrest [29]. Intranasal delivery of NGF to the brain is a non-invasive and safe route to achieve a significant and effective concentration of NGF in selected brain areas [30]. Based on these background and scientific evidences, we conduct a new clinical investigation further exploring the effects of intranasal hr-NGF administration on brain functions of 3 children with post-traumatic UWS [2, 30].

## Materials and methods

### Study design and eligibility

In this prospective interventional study children, aged 3 to 10 years, with post-traumatic UWS were enrolled. These patients showed spontaneous eye opening and independent vital functions but they cannot functionally communicate their thoughts or feelings and appear completely unaware of their surroundings and themselves, as defined in the most recent literature about UWS [31]. In these children, the post-traumatic UWS had to be stabilized and unresponsive to any documented treatment at least 6 months after the TBI, based on clinical, neuroradiological and instrumental data [32]. Exclusion criteria were the active presence of intraparenchymal hemorrhages, intraventricular hemorrhages, subdural hematomas, epidural hematomas, and known hypersensitivity to the medical principles or excipients at the moment of enrollment. Parents were asked to sign written informed consent before starting the therapy. The study was approved by the Ethic Committee (approval n° 5169/20, ID 2989) of the Fondazione Policlinico Universitario Agostino Gemelli – IRCCS, Rome (Italy). This study is also a registered clinical trial (EudraCT number 2019-002282-35).

### Study procedures

Patients underwent intranasal administration of hr-NGF (Oxervate by Dompè Farmaceutici, Milan, Italy) in four cycles of seven days each. Neuroradiological evaluation by Positron Emission Tomography scan (PET), Single Photon Emission Computed Tomography (SPECT), Magnetic Resonance Imaging (MRI), Electroencephalography (EEG), and Power Spectral Density (PSD) of the brain was preliminarily performed. These exams were completed at baseline and one month after the end of the treatment. The main aim of the study was to investigate any changes in patient’s neurological and clinical conditions and to detect variations in neuro-radiological and electrophysiological findings at the end of the therapy.

### Intranasal hr-NGF administration

Intranasal clinical grade hr-NGF aqueous solution at a total dose of 50 μg/kg was administered to each patient. The product used was OXERVATE, consisting of cenegermin-bkbj (hr-NGF) ophthalmic solution 0.002% (20 µg/mL) (Dompè Farmaceutici SpA, Milano, Italy). The vial of Oxervate was stored at 4 °C until their usage. The total dose of hr-NGF (50 µg/kg) was divided in four cycles of treatment. In each cycle ¼ of total dose was distributed in 21 doses and each dose, divided in the 2 nostrils, administered 3 times a day for 7 consecutive days. The cycles of administration started on day 1, 31, 61 and 91 of treatment. In this way the dose and final volume of each one administration was calculated according with the patients’ weight. “MAD Nasal™ (Intranasal Mucosal Atomization Device from Teleflex”—MAD100, Version Model Number IPN048826), was used to deliver hr-NGF, without any process of dilution or lyophilization, via a fine spray (30 micron), in order to facilitate its absorption by olfactory and trigeminal nerve fibers. In literature it is demonstrated a significant increase, after intranasal administration, of NGF concentration in the entrance areas to the brain parenchyma (olfactory bulbs and brainstem) and significantly higher concentrations of this neurotrophin than the basal ones in almost the entire brain parenchyma [30, 33]. To avoid drug absorption interference, before the administration of hr-NGF, nostrils were washed with 1 ml of saline solution and then aspirated.

### Neurological assessment

Neurological assessment was achieved adopting the gross motor function measure and the Modified Ashworth Scale (Additional file 1: eFigure S1 and Additional file 2: S2); these evaluations were scheduled at baseline and at the end of therapy. The gross motor function measure (GMFM) is an observational clinical tool developed to quantitatively assess the changes in motor function in children with cerebral palsy [34, 35]. It has been proven to be valid and reliable for analyzing the motor skills of pediatric patients affected by cerebral palsy and other brain damage undergoing rehabilitation. Besides, the Gross Motor Function Classification System (GMFCS) was used to assess each patient’s level of gross motor function with skill levels from I to V (Additional file 3: eFigure S3) [35, 36]. The Modified Ashworth Scale is a validated clinical tool to measure the increase of muscle tone and to determine the efficacy of the treatments in patients with spasticity [37, 38]. Moreover, the study investigates the possible changes in quality of life of patients enrolled after the treatment, according to the Disability Rating Scale (DRS, Additional file 4: eFigure S4). This scale was created to evaluate the effects of severe TBI to determine how long recovery might take [39]. This tool was used for the clinical assessment of the patients before and after the treatment, working with parents and caregivers.

### Safety assessment

Medical history and physical examination were recorded for each patient at baseline, at the beginning of every treatment cycle and one month after the end of the therapy. We evaluated safety detecting adverse events related to the treatment. Clinical and neurological changes during the hr-NGF administration were reported. The therapy tolerability was evaluated both by investigators and caregivers.

### Neuroradiological assessment

PET, SPECT, EEG, and PSD were performed in all enrolled children for the neuroradiological assessment. The technical details of each one of these exams are described in Additional file 5

## Results

In this study we enrolled 3 children with post-traumatic UWS. All children presented, at the admission in the Pediatric Intensive Care Unit (PICU), a glasgow coma scale (GCS) of 4 and their hospitalization was about 1 month in the PICU and 5 months in the Neurorehabilitation Unit based on their clinical and neurological conditions.

### Case#1

**Fig. 1:**
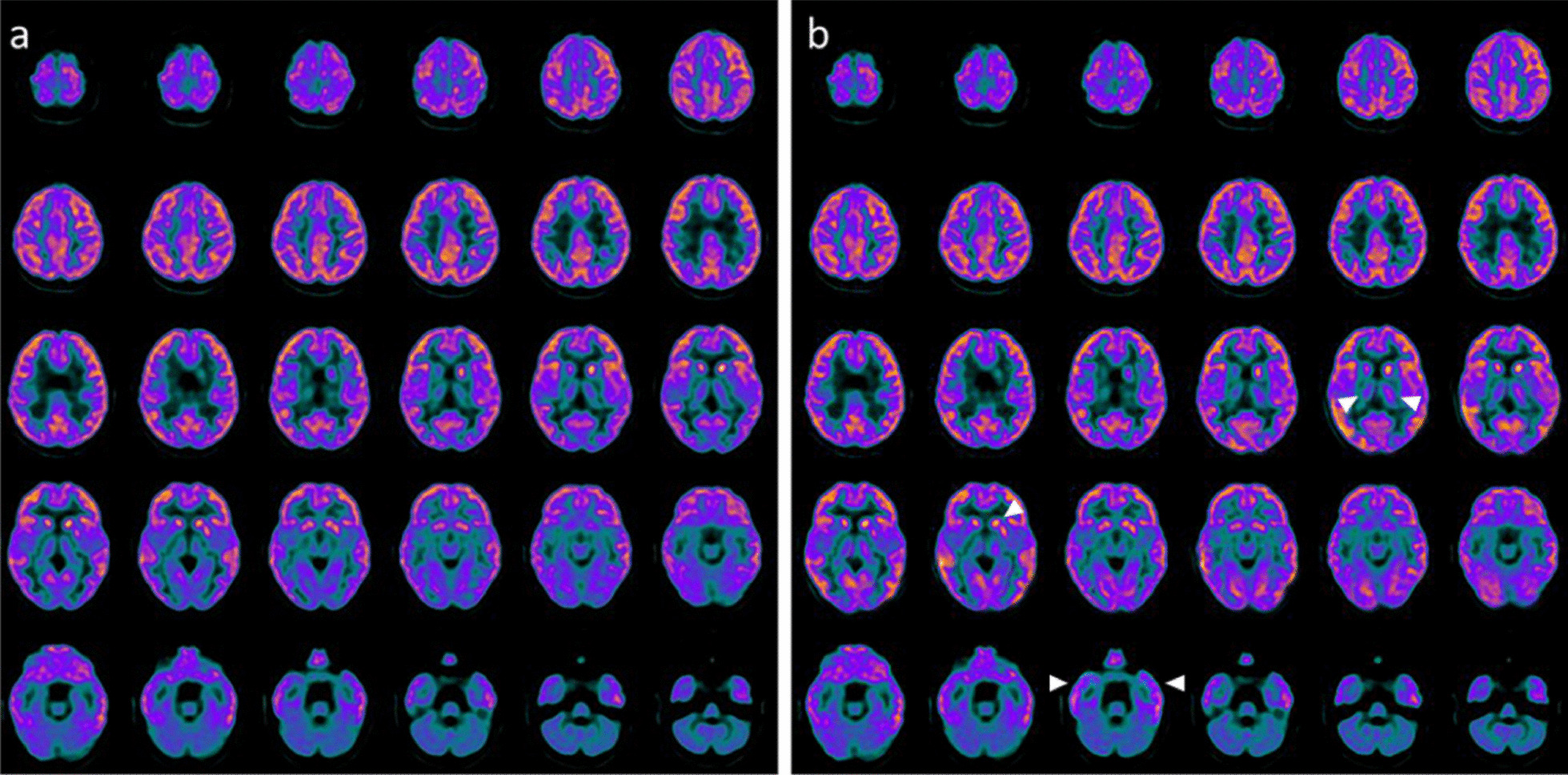
PET before and after the treatment with hr-NGF. **a, b**: Brain 18F-FDG PET axial slices performed before (**a**) and after **b** intranasal hr-NGF treatment. A mild global reduction in 18F-FDG uptake was observed in all cortical regions, whereas a more marked reduction was detected in all subcortical regions (**a**). After hr-NGF administration, an increase in radiotracer uptake was found in the bilateral temporal cortex (right: + 7%; left: + 7%), right and left thalamus (+ 6% and + 4%, respectively) and the left caudate nucleus (+ 9%) (**b**)

**Fig. 2:**
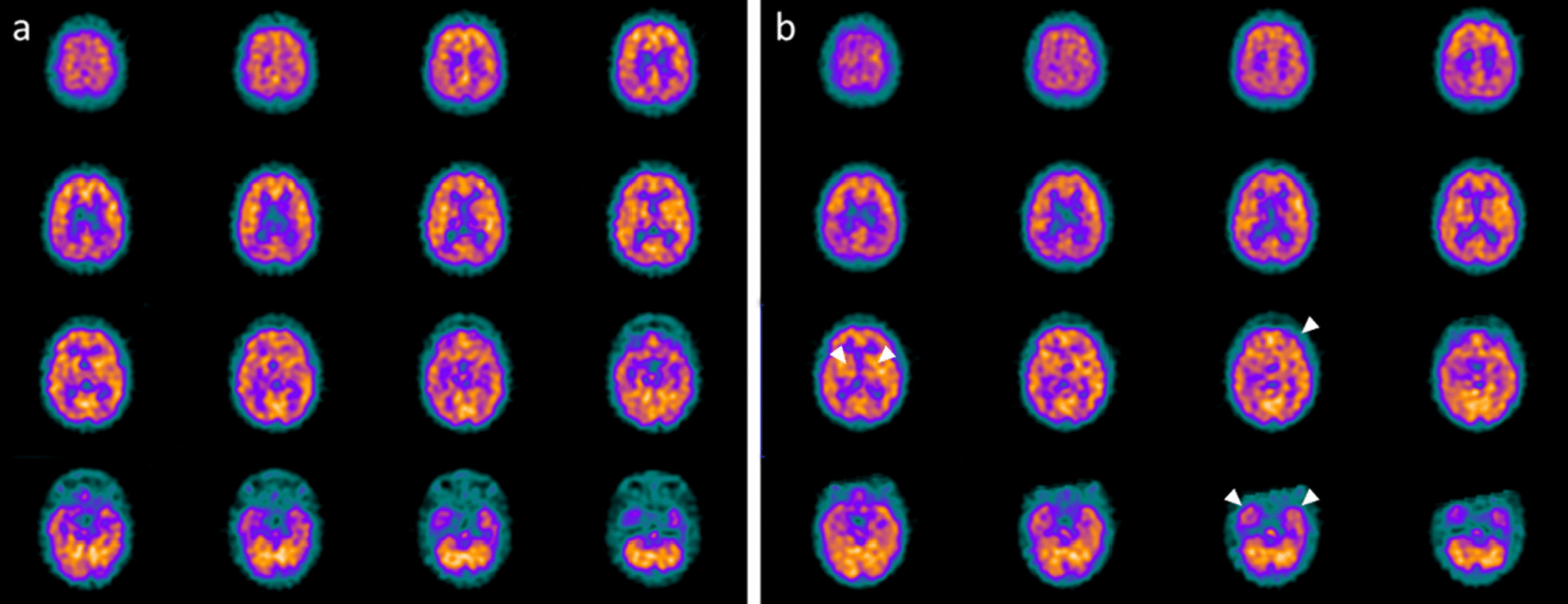
SPECT before and after the treatment with hr-NGF. **a, b**: Perfusion SPECT images before (**a**) and after **b** intranasal hr-NGF administration. ^99m^Tc-HMPAO SPECT images (transaxial slices) before hr-NGF treatment showed a mild reduction in radiotracer uptake (hypoperfusion) in the right and left parietal cortices, left frontal cortex, right temporal cortex, left temporal pole, as well as in the caudate nucleus, putamen and thalamus, bilaterally (**a**). After hr-NGF treatment, a slight increase in ^99m^Tc-HMPAO uptake was detected in the left frontal cortex (+ 10%), right temporal cortex (+ 9%), left temporal pole (+ 13%), right and left caudate nucleus (+ 10% and + 11%, respectively), right and left putamen (+ 17% and + 15%, respectively), right and left thalamus (+ 11% and + 10%, respectively) (**b**)

This patient was admitted at the age of 4 years at the Pediatric Intensive Care Unit (PICU) after a severe TBI secondary to a car accident. He underwent immediate resuscitation maneuvers and tracheal intubation. At admission in the PICU, the first neurologic examination showed a glasgow coma scale (GCS) of 4. Brain MRI showed deep and diffuse hemorrhagic petechiae, multiple frontal and temporal cortico-subcortical biemisferic hemorrhagic contusions, signs of axonal distraction at brainstem and at the splenium of the corpus callosum. Bilateral areas of increased T2 signal involving basal ganglia consistent with anoxic injury component were also detected (data not shown). After 7 days from the trauma the sedation was stopped and the child showed eye opening with a complete failure of respiratory trigger, so the patient underwent tracheostomy for mechanical ventilation and a placement of a gastrostomy tube. Six months after TBI the child showed a lack of swallowing, inability to speech, reflex movements without response to command and minimally conscious state in the presence of post-traumatic UWS. The neurological examination showed an alert and conscious child, severe communicative and neuropsychological impairment and complete dysphagia. The patient was fed only by gastrostomy. An oral-motor dyspraxia was also detected and only reflexed pattern movements were present. Increased muscle tone was observed, especially in the lower limbs with concomitant signs of spasticity. A targeted management of spasticity with botulinum toxin injection in specific muscle groups was performed without any improvement. The patient also received numerous physiotherapy cycles without any evidence of improvement in his communicative and cognitive skills and in his motor functions. Six years after TBI (at the age of 10 years), due to the persistent of post-traumatic UWS, treatment with intranasal hr-NGF administration was started, based on the schedule reported in Material and Method section. After NGF treatment, significant improvements were observed in some cognitive processes, mainly in the planning of a communication strategy, attention and verbal comprehension. At the end of intranasal NGF administration, the patient showed a substantial amelioration also in facial mimicry and in communicating by eyelids closure. The child was also able to eat from the mouth; in fact, some improvements were also observed in oral motility. The patient had a better relationship with his family members and all the caregivers and was less frustrated and in a better mood. Oral motor dyspraxia progressively improved too, with enhanced oral motility control including mouth opening, tongue motility, mastication and swallowing. The ability to feed also improved and the child became able to eat little amounts of food with a parent-reported better discrimination regarding taste. In association with the improvement in oral motor dyspraxia, other acquired skills, included phonation with more explicit emission of sounds, were observed. Some hand finger movements during play with characteristics of voluntary control have been observed and even improved muscular tone and tropism, with a significant amelioration of his spasticity. The child also reacquired the cough reflex and hiccups, previously absent. Based on the protocol, the child was subjected to PET, SPECT, EEG, and PSD before and after the treatment with hr-NGF. The first PET and SPECT pointed out a global reduction in tracer uptake at the cortical, subcortical and cerebellar levels (Figs. 1a and 2a, respectively). At the end of the treatment, both PET and SPECT showed an increase in tracer uptake in specific brain areas, such as in the bilateral temporal cortex, thalamus, left caudate nucleus and cerebellum. Figures 1b and 2b reported all detailed descriptions of neuroimaging modifications after the treatment. EEG recording performed before the beginning of hr-NGF treatment showed severe and diffuse low-voltage background activity. The EEG examination carried out after the end of NGF treatment showed an improvement in the electrical cerebral activity, mainly in the anterior regions with diffuse rhythms, while topographical analysis of the PSD distribution of the EEG signal documented a reduction in the slow frequency bands (delta and theta) in post treatment records, a more modest reduction in the alpha band, and an increase in the fast band activities (beta). These changes had a different distribution, as highlighted in Figures @@7a–e. These improvements of functional (PET/CT and SPECT/CT) and electrophysiological (EEG and PSD) findings, were confirmed by a concomitant amelioration of all the scales used to evaluate the neurological and clinical conditions of treated children. In particular, the mean GMFM pre-treatment was 2.78% (V level for GMCS for infant cerebral palsy). An improvement of GMFM of 22% was evidenced after the treatment, while also Ashworth scale showed an improvement in spasticity for ankles and lower limbs (from 3 to 2 scores). In addition, according to the DRS, this patient collected an initial score suggestive of a Vegetative State (22–24 points of the DRS). After the treatment with hr-NGF the child gained 4 points in this scale—thanks to a better communication strategy and motor response—going from Vegetative State to Severe Disability (17–21 points of the DRS)**.** No adverse effects were reported during the study period.

### Case#2

**Fig. 3:**
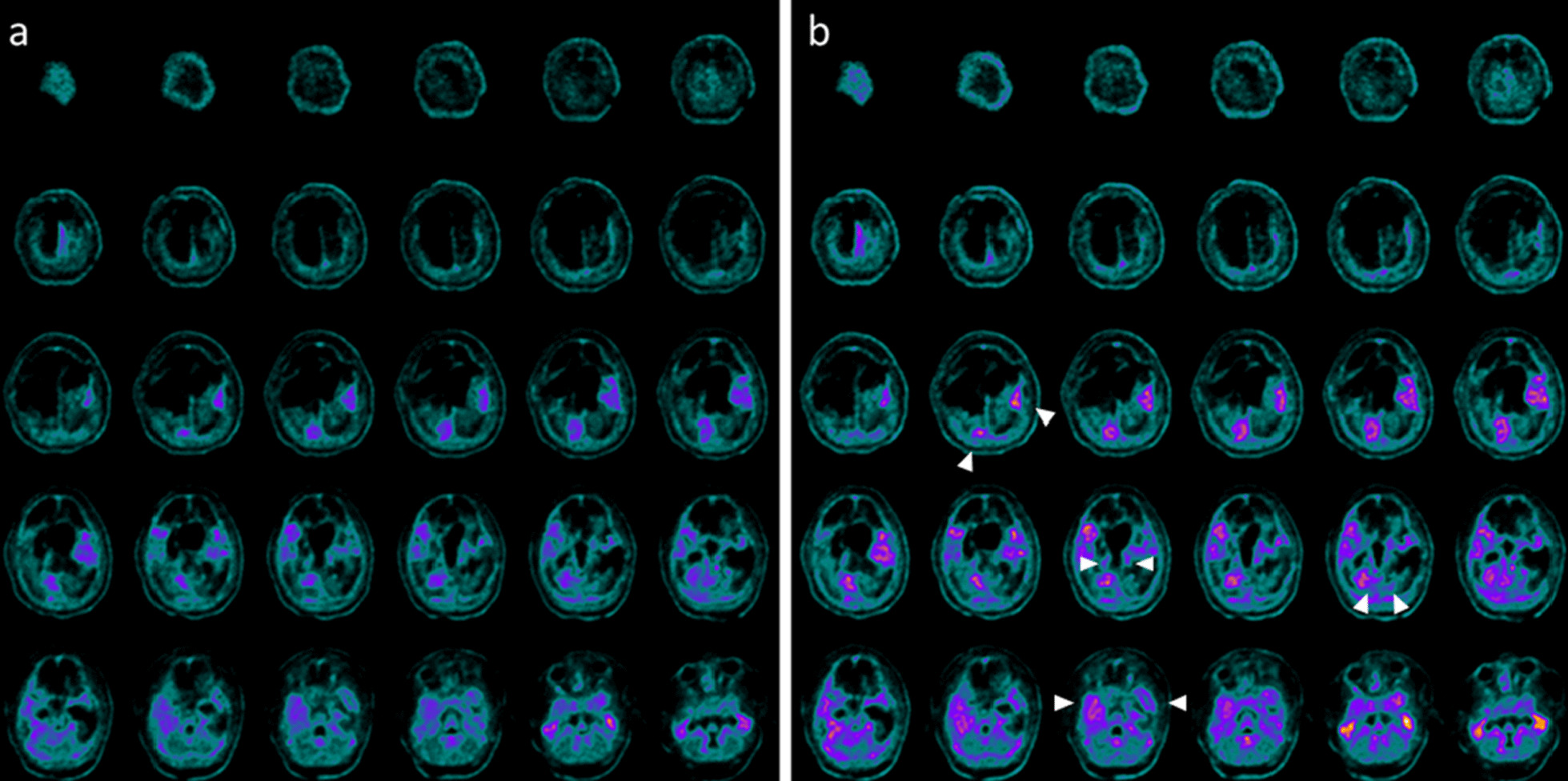
PET before and after the treatment with hr-NGF. **a, b**: Brain 18F-FDG PET axial slices performed before (**a**) and after **b** hr-NGF treatment. A severe global reduction in 18F-FDG uptake was observed in all cortical and subcortical regions as well as in the cerebellum (a). After hr-NGF administration, an increase in radiotracer uptake was detected in the left and right temporal cortex (right: + 18%; left: + 15%), bilateral parietal cortex (right: + 18%; left + 15%), right and left occipital cortex (right: + 10%; left: + 13%) and right and left thalamus (+ 7% and + 8%, respectively) (**b**)

**Fig. 4:**
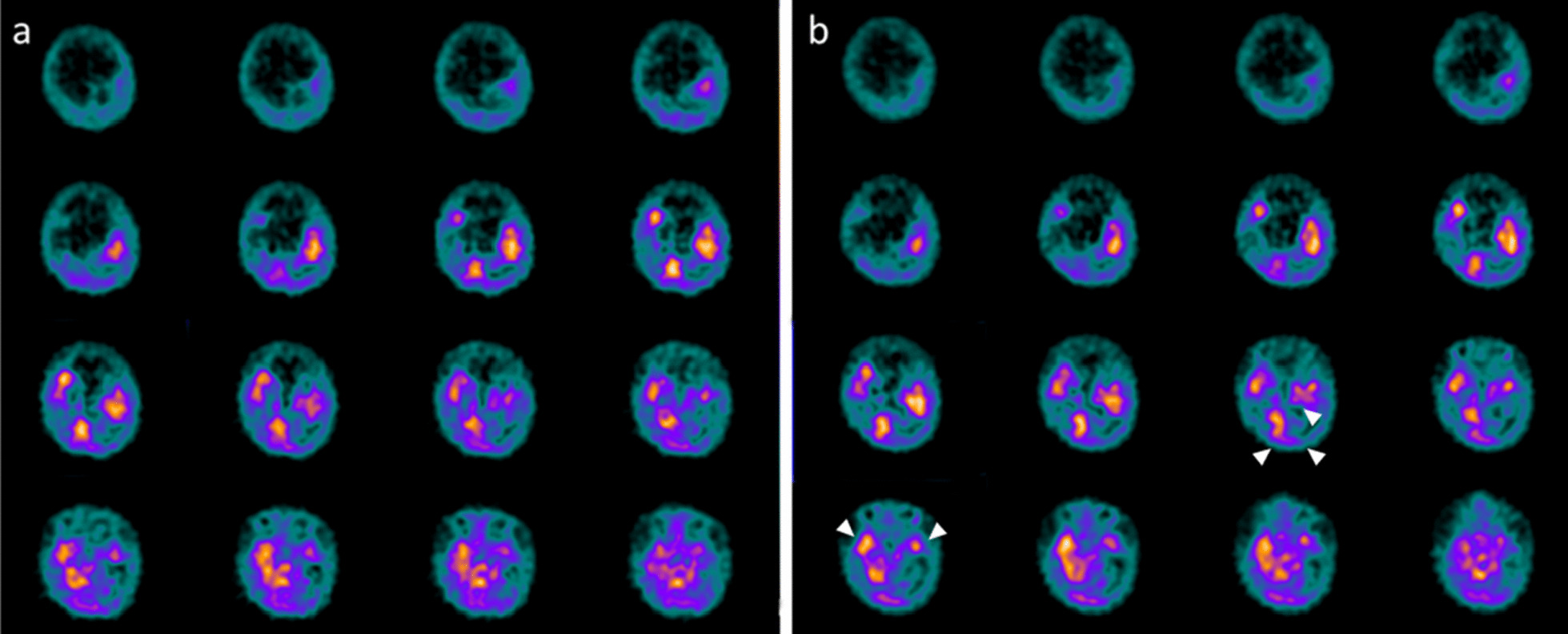
SPECT before and after the treatment with hr-NGF. **a, b:** Perfusion SPECT images before (**a**) and after **b** hr-NGF administration. ^99m^Tc-HMPAO SPECT images (transaxial slices) before hr-NGF treatment showed a severe reduction in radiotracer uptake (hypoperfusion) in almost all cortical and subcortical areas as well as in the cerebellum (**a**). After hr-NGF treatment, an increase in ^99m^Tc-HMPAO uptake was found in the left temporal cortex (+ 20%), right anterior temporal cortex (+ 13%), left thalamus (+ 10%) and bilateral occipital cortex (right: + 14%; left: + 18%, respectively) (**b**)

A 17 old-month girl was admitted at our PICU after a severe crushing head injury by motor vehicle accident. At admission, the neurologic examination showed a glasgow coma scale (GCS) of 4. Brain MRI showed blood share in the lateral ventricles, deep and diffuse hemorrhagic petechiae, multiple frontal, temporal and occipital cortico-subcortical hemorrhagic contusions, signs of diffuse axonal injury at brainstem, at the splenium of the corpus callosum, involving nucleocapsular and midbrain regions (data not shown). After 6 days from admission, the sedation was stopped and the child showed a relative alert state. Due to the presence of respiratory failure, the patient underwent tracheostomy and a gastrostomy was placed to ensure appropriate nutrition. In addition, the patient developed central diabetes insipidus, panhypopituitarism, mild hyposurrenalism and impaired thermoregulation with tendency to hypothermia and bradycardia. At day 75 after TBI the child showed a lack of swallowing, inability to speech, reflex movements without response to command and minimally conscious state in the presence of post-traumatic UWS. The neurological examination showed an alert and conscious child with severe communicative and neuropsychological impairment and complete dysphagia. Increased muscle tone was observed, especially in the upper and lower limbs, with initial signs of spasticity, not responding to botulinum toxin injection. Thirty-one months after TBI (at the age of 4 years), due to the persistent of post-traumatic UWS, treatment with intranasal hr-NGF administration was started. At the end of intranasal NGF administration, the patient showed a significant improvement in facial mimicry. The child was also able to eat from the mouth; in fact, some improvements were also observed in oral motility and head rotation. Oral motor dyspraxia progressively improved too, with enhanced oral motility control. In association with the improvement in oral motor dyspraxia, other acquired skills, included phonation were observed. Moreover, during the cycles of hr-NGF therapy, a progressive but constant enhancement of head movements (mainly in head lateral rotation and minimally in up and down movements) was reported. Some hand finger movements have been observed with improved muscular tone, tropism, and spasticity. A recovery of some hypothalamic functions, such as an improvement in thermoregulation, cardiac rhythm, and in the sleep–wake cycle, were also detected. The child underwent PET, SPECT, EEG, and PSD before and after the treatment with hr-NGF. The first PET and SPECT pointed out a marked and severe reduction in tracer uptake at the cortical, subcortical and cerebellar levels (Figs. 3a and 4a). At the end of the treatment, both PET and SPECT showed a remarkable increase in tracer uptake in specific brain areas, such as bilateral temporal, parietal, and occipital cortex, thalami and cerebellum. Figures 3b and 4b reported these modifications after the treatment with hr-NGF. EEG recording performed before the beginning of hr-NGF treatment showed severe low-voltage background activity, with sporadic theta-delta activity in the right and left fronto-temporal and occipital regions. The activity was markedly and diffusely depressed with a poor electric organization. The EEG examination carried out at the end of NGF treatment showed an improvement in the electrical cerebral activity: a quantifiable 4–5 Hz background theta-delta activity is evident bilaterally on the anterior and posterior regions, intermixed with abundant diffuse rapid rhythms. Topographical analysis of the PSD distribution of the EEG signal documented a reduction in the PSD of the slow frequency bands (delta and theta) in post treatment records, a more modest reduction in the PSD of the alpha band, and an increase in the fast band activities (beta). These changes had a different distribution, as highlighted in Figures @@7a–e. All these clinical and neuroradiological findings were confirmed by the GMFM scale that increased from 3 to 6.8% and by the Ashworth scale that highlighted an improvement in spasticity for the right side of the lower and upper limbs (3 points). Regarding the assessment of the degree of disability, the total DRS was 24 before the treatment, indicative of a vegetative state. The areas of greatest impairment were autonomy, ability to perform personal activities and employability. After the treatment, a 3-point improvement on this scale was observed with the switch from a vegetative state to a severe disability (21 points of the DRS). No side effects were reported during the study period.

### Case#3

**Fig. 5:**
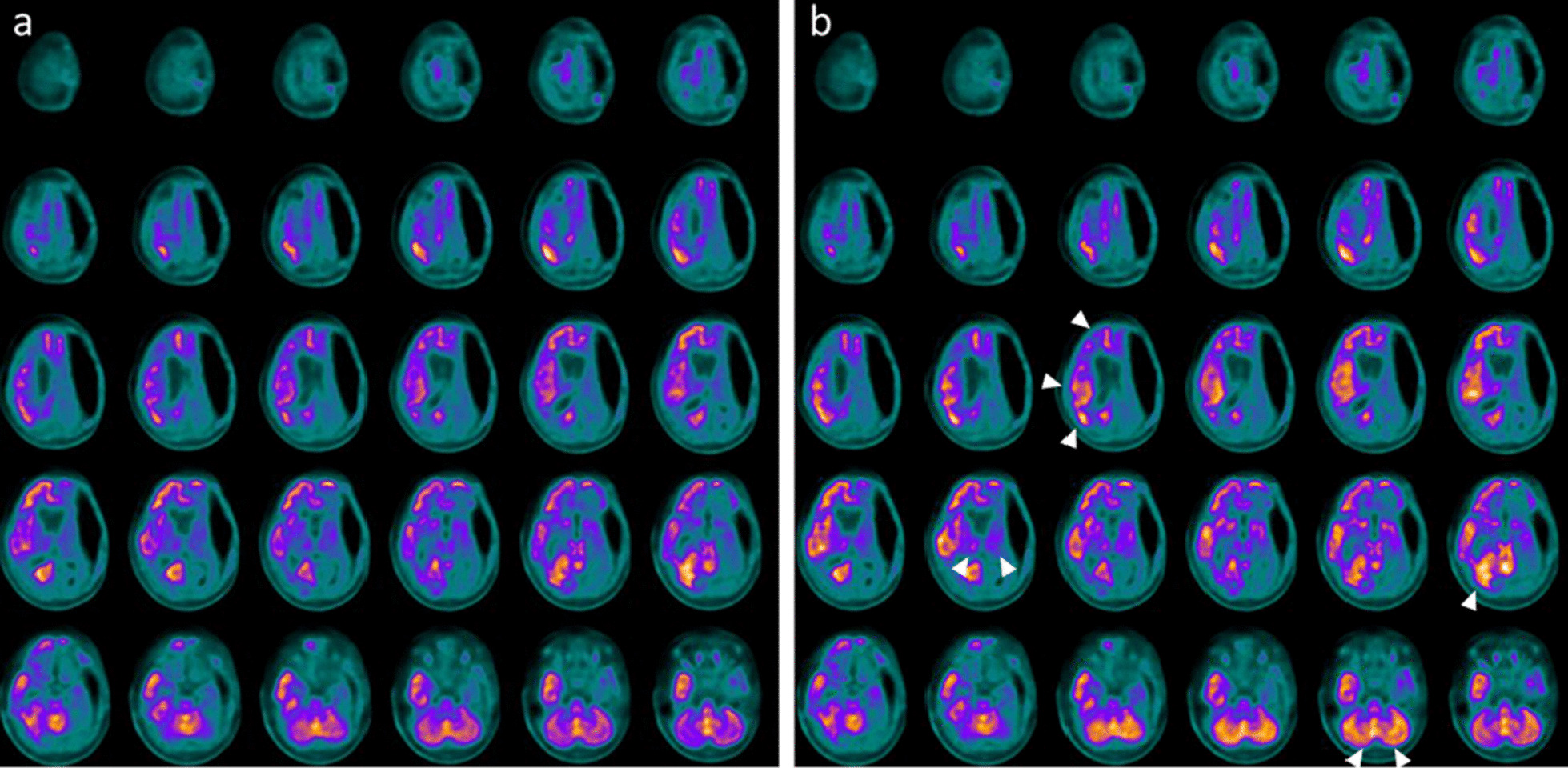
PET before and after the treatment with hr-NGF. **a, b:** Brain 18F-FDG PET axial slices performed before (**a**) and after **b** hr-NGF treatment. A severe reduction in 18F-FDG uptake was observed in all cortical and subcortical regions of the left hemisphere, whereas a mild reduction was detected in the whole cerebellum and all cortical and subcortical regions of the right hemisphere (**a**). After hr-NGF administration, an increase in radiotracer uptake was found in the right frontal cortex (+ 11%), right temporal cortex (+ 15%), right parietal cortex (+ 14%), right occipital cortex (+ 22%), right and left thalamus (+ 10% and + 7%, respectively) and cerebellum (+ 33%) (**b**)

**Fig. 6:**
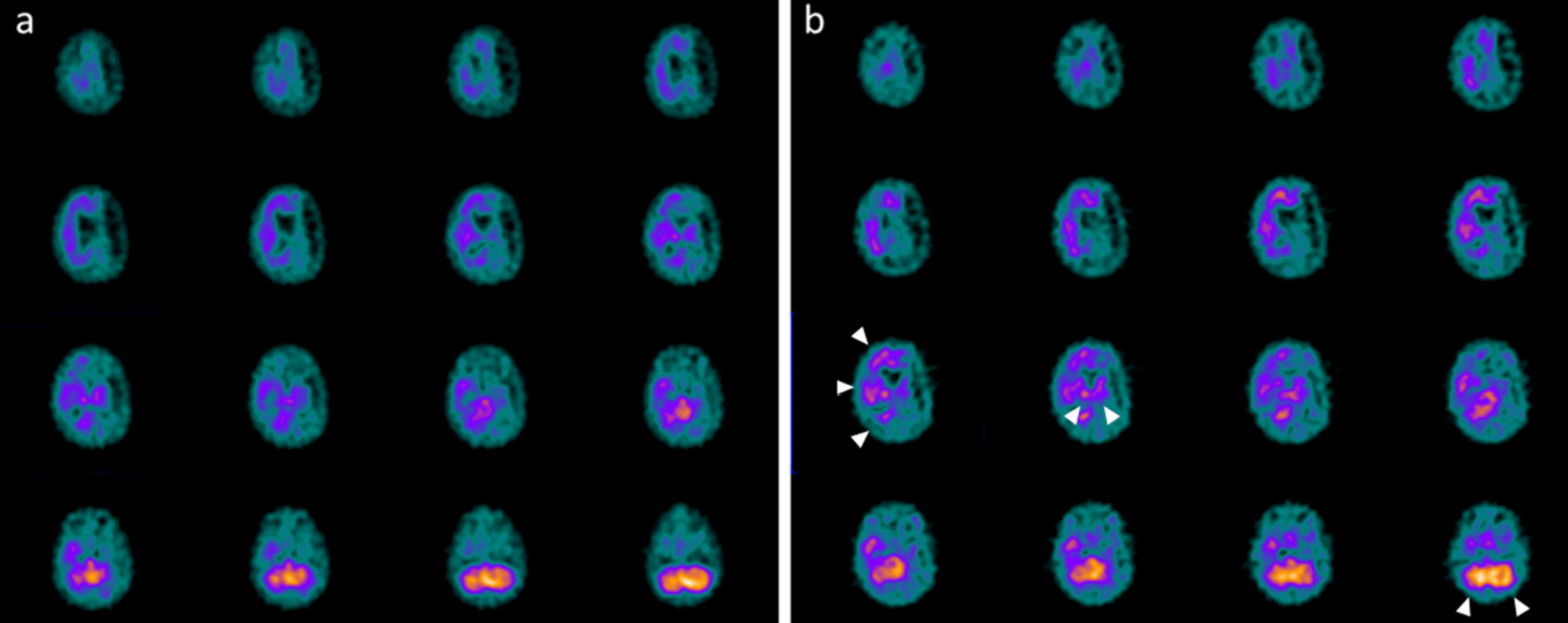
SPECT before and after the treatment with hr-NGF. **a, b:** Perfusion SPECT images before (**a**) and after (**b**) hr-NGF administration. ^99m^Tc-HMPAO SPECT images (transaxial slices) before hr-NGF treatment showed a severe reduction in radiotracer uptake (hypoperfusion) in all cortical regions of the left hemisphere. A moderate-to-severe reduction in ^99m^Tc-HMPAO uptake was observed in the left subcortical areas, right cortical regions as well as right subcortical areas, whereas a mild reduction was detected in the cerebellum (**a**). After hr-NGF treatment, an increase in radiotracer uptake was found in the right frontal cortex (+ 16%), right temporal cortex (+ 20%), right parietal cortex (+ 14%), right occipital cortex (+ 26%) right and left thalamus (7.5% and 9%, respectively) and cerebellum (+ 34%) (**b**)

**Fig. 7:**
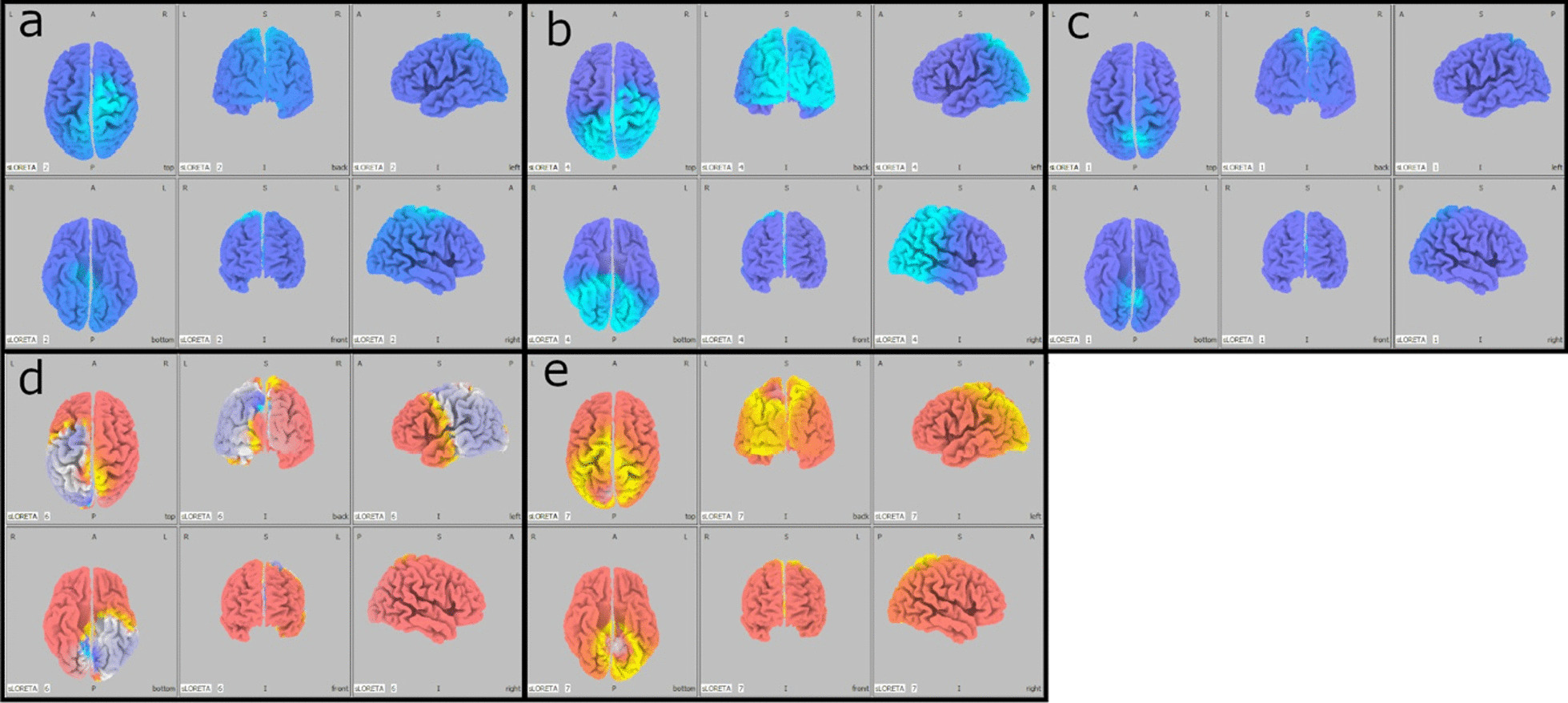
Power Spectral Density. **a–e.** Comparison of topographic distribution of EEG Power Spectral Density before vs after intranasal NGF administration showed a mean cumulative increase of slow and organized rhythms at the expense of fast and disorganized ones. Blue coloring corresponds to a better expression of waves in post treatment EEG, despite to a red shades that indicate a wider expression in pretreatment pathways. In particular: diffuse increase of theta activity, mainly evident on the midline regions **(a)**; diffuse increase of alfa activity, particularly evident on the parieto-occipital regions **(b)**; improvements in delta waves mainly in Broadman areas and limbic region with cingulum zone **(c);** diffuse decrease of beta activity, which does not occur on the left on the parieto-occipital regions **(d)**; diffuse decrease of gamma activity, particularly evident in parietal-midline areas **(e)**

A 14 old-month girl was admitted at the PICU after a severe TBI secondary to a car accident. At admission the neurologic examination showed a glasgow coma scale (GCS) of 4. Brain MRI showed diffuse brain edema, hemorrhagic petechiae in the grey matter, multiple frontal and temporal cortico-subcortical hemorrhagic contusions, signs of diffuse axonal injury at brainstem, and thinning of cerebral circumvolutions (data not shown). After 7 days the sedation was stopped and the child evidenced a failure of respiratory trigger, so the patient underwent tracheostomy and gastrostomy. After about 3 months from TBI the patient showed inability to speech, reflex movements without response to command and minimally conscious state in the presence of post-traumatic UWS. Communication was possible only through eye movements. Somatosensory evoked potentials (SSEP) showed a marked distress functional tract of the upper and lower limbs. The child also presented a spastic-dystonic tetraparesis and developed an epileptic encephalopathy requiring appropriate therapy. At this time, the neurological examination showed an alert and conscious child with severe communicative and neuropsychological impairment. The child also lost bladder and bowel functions; an oral-motor dyspraxia was also detected with the presence of loss of skin sensitiveness, while a progressive increase of muscle tone, especially in the upper and lower limbs, was noticed. Due to persistence of increased muscle tone and spasticity, a targeted management of spasticity with botulinum toxin injection was performed without any improvement. Ten months after TBI, at the age of 2 years, due to the persistent of post-traumatic UWS and after proper and standardized medical, neuro-intensive and rehabilitative care, treatment with intranasal hr-NGF administration was started. After NGF treatment, significant improvements were observed in some cognitive processes, mainly in attention and verbal comprehension. At the end of intranasal NGF administration, the patient showed an improvement in the capacity to knit eyebrows, curl nose, crying with tears, major symmetry in the smile and more meaning to eyelids closure in communicating. The child was also able to eat from the mouth; in fact, some improvements were also observed in oral motility and head rotation. Due to these neurological and clinical modifications, an alternative and increasing communication program, involving all the family members, was started with great success. Oral motor dyspraxia progressively improved too, with enhanced oral motility control including mouth opening, tongue motility, mastication and swallowing. The ability to feed also improved and the child became able to eat little amounts of food. In association with the improvement in oral motor dyspraxia, other acquired skills, included phonation with more explicit emission of sounds, were observed. Moreover, during the cycles of NGF therapy, a progressive but constant enhancement of head movements (mainly in head lateral rotation and minimally in up and down movements) was reported. The child responded more effectively to visual stimuli, fixating and tracking the targets. She managed to keep her eyes open and to recognize familiar voices, turning her head towards the source of the sound. The left deviation of her eyes was no longer present and her eyes were well-positioned on the midline; horizontal nystagmus ameliorated and was present only after position changes. The corneal, vestibulo-ocular, and inducible cough reflexes, which had previously been absent, were also restored. Another important amelioration was represented by the improvement in bowel function, losing the need for stimulation to evacuate. We observed also an increased tolerance to passive mobilization, with a significant reduction of muscular hypertone and trophism, spasticity and dystonic seizures. According to these clinical and neurological modifications, also GMFM total score increased from 3.4 to 6.6% after the treatment, while a significant improvement in spasticity (assessed by modified Ashworth Scale) of 4 points was reported, with progressive reduction of muscle hypertonus**.** Moreover, the DRS collected an initial score suggestive of an extreme vegetative state (25–29 points). At the end of the treatment, there was an improvement of 4 points in her DRS, with a consequent change of category from Extreme Vegetative State to Severe Disability (17–21 points). PET and SPECT, before the treatment, pointed out a marked and global reduction in tracer uptake at the cortical, subcortical, thalami, and cerebellar levels (Fig. 5a and 6a). At the end of hr-NGF administration, PET and SPET showed a remarkable increase in radiotracer uptake in the right frontal, temporal, parietal and occipital cortex, right and left thalamus and cerebellum (Figs. 5b and 6b). Figures 5 and 6 highlighted the modifications of neuroimaging before and after the treatment. EEG recording performed before NGF treatment showed severe low-voltage background activity. Sporadic theta-delta activity in the right fronto-temporal regions could be appreciated. Sleep was scarcely distinguished from wakefulness and the activity was markedly and diffusely depressed. The EEG examination carried out at the end of NGF treatment showed an improvement in the electrical cerebral activity: a quantifiable 4–5 Hz background theta-delta activity is evident bilaterally on the anterior regions (right > left), intermixed with abundant diffuse rapid rhythms. Topographical analysis of the PSD distribution of the EEG signal showed a mean cumulative increase of slow and organized rhythms at the expense of fast and disorganized ones. These changes had a different distribution, as highlighted in Fig. 7a–e**.** No adverse effects were described during the study period.

## Discussion

TBI is the leading cause of morbidity and mortality among children and young adults [40]. Children with severe TBI are more likely to develop clinical, neurologic and behavioral problems [41]. Severe TBI shows a high mortality rate: about 25% of severe TBI patients do not survive, and the majority of survivors present varying degrees of disability [42]. Clinical outcome in TBI patients depends on both by the primary injury and by secondary brain damage [43]. The primary injury involves tissue damage and disruption caused by the mechanical force of impact. Secondary brain injuries begin to occur within several minutes of primary injury and persist for days to months following the primary injury [43]. Secondary brain damage expands progressively and centrifugally, culminating in neuronal cell death. Typically, neurons damaged in the primary injury undergo necrotic cell death, while neurons that succumb to secondary injury faced apoptotic cell death. The traumatic penumbra surrounds the primary lesion and represents the portion of damaged brain tissue that can potentially be regenerated [44]. Neurogenesis and neuronal repair are high during the first month after brain injuries and decreases thereafter [45–47]. Although low, persistent neurogenesis has been described until four months after ischemic stroke and severe TBI [48]. Only a few experimental studies characterized and quantified the volume of repaired brain tissue after injury, but some of them have described that only 0.2% of neurons are replaced by spontaneous neurogenesis after brain damage, since most of new mature neurons do not survive. Based on these studies, traumatic penumbra is a crucial therapeutic target for many forms of brain injury. Glial cells are first activated to protect neurons in the penumbra, proliferating and secreting anti-apoptotic molecules, such as NGF and other neurotrophic factors [49–51]. NGF supports neuronal growth, survival, and differentiation [52], restoring the function of injured neurons [6], and has been investigated as a novel treatment strategy both in experimental animal models and in TBI patients.

There are several hypotheses to explain why NGF exerts beneficial effects in traumatic brain injury [53, 54]. Preclinical and clinical data obtained to date support the therapeutic effect of NGF on brain cells acting on primary and secondary damage induced by trauma. Specifically, it has been demonstrated a promising action of NGF on brain cells population that are primarily injured by mechanical insult and that trigger neuronal death, neuroinflammation, mitochondrial failure and axonal degeneration. The inhibition by NGF of these early TBI pathological events are crucial to reduce the probability of severe secondary damage that represent one of the main causes of permanent motor and cognitive disability in TBI patients [12]. NGF has also shown to be effective in clinical setting improving brain metabolism and motor function in children with chronic brain damage, showing a relevant pharmacological actions on metabolic activity of brain cells [27–29].

All these data led us to continue exploring the pharmacological potential of NGF in patients affected by severe pathological dysfunction induced by TBI. Furthermore, it is also relevant to highlight the type of administration that we used to deliver this neurotrophin in to the brain. Intranasal delivery of NGF via the olfactory bulb and the trigeminal nerve pathways, leads to substantial concentrations of this neurotrophin into the brain [55], mainly in the frontal and parietal cortices, thalamus, cerebellum and striatal level [56], which are most often affected by stroke and TBI. At the striatal level, one of the possible targets of NGF could be cholinergic interneurons expressing TrkA receptor in detectable amounts, both during development and in adulthood [57, 58]. Alterations of brain cholinergic neurons have been reported as a consequence of brain trauma and ischemia [59], and striatal cholinergic dysfunction is associated with various forms of dystonia and spasticity [60], which are common long-term neurological sequelae in patients suffering from severe TBI [61].

Delivering neurotrophic factors into the brain has classically been a significant challenge owing to the presence of the blood–brain and blood–cerebrospinal fluid barriers limiting drug penetration into the CNS [20, 62].

Accumulated experiences have pointed to the existence of a direct pathway from the nose to the brain that can facilitate the release of drugs into the CNS. The pharmacology of intranasal NGF seems to be heading towards promising development, based on the ease of administration, the efficiency of drug distribution to the brain parenchyma and the efficacy demonstrated in a number of preclinical studies [63, 64].

In humans, intranasal delivery of NGF has been recently investigated both in a child with severe TBI and in 3 children with chronic vegetative state secondary to prolonged out-hospital cardiac arrest, with a significant improvement both of their cognitive and motor functions after the treatment [27, 29].

Based on these previous experiences, in this study we report, for the first time, the neuroprotective effects of hr-NGF in 3 children affected by post-traumatic UWS. Despite advanced and up-to-date critical, neuro-intensive and rehabilitative care, these patients showed no neurological improvement after long time from TBI (from 10 months to 6 years), so we decided to start an experimental treatment with intranasal hr-NGF administration. This new therapeutic approach was followed by a relevant improvement of functional (PET/CT and SPECT/CT) and electrophysiological (EEG and PSD) results, with a concomitant amelioration of the patients’ clinical and neurological conditions, characterized by the acquisition of voluntary movements of their legs, arms and fingers, improved facial mimicry and phonation, small grimaces and little smiles, attention and verbal comprehension, with consequent better interaction with their parents and caregivers, as testified by the changes in GMFM and DRS. After NGF treatment, these children recovered the ability to cry, cough reflex, control of oral motility and feeding capacity, showing an improvement also in bowel and urinary functions. Moreover, the most important clinical improvement to highlight in all these patients is the significant reduction of their muscle hypertone and spasticity, as reported by the modifications of Ashworth Scale. This result correlates well with the increased uptake in glucose metabolism and vascularization in the cerebellum and thalamus bilaterally and with the improvement of brain perfusion in the same areas. Both these changes have been documented by PET and SPECT investigations at the level of the two aforementioned structures and by the improvement in brain electric activity, documented via PSD, at the level of Brodmann’s areas, which is in communication with the thalamus. To this regard, it is important to mention that muscle tone is closely related to these two structures: on one hand the cerebellum which actively participate in the regulation of posture and muscle tone and, on the other hand, the thalamus which is in communication with the premotor and supplementary motor cortex, with an established connection to both voluntary and non-voluntary movements. Neuroradiological, electrophysiological, and clinical changes observed in these children have been so remarkable and occurred in a relatively short time (about one month after the end of therapy) that is conceivable they are related to the neuroprotective effects of the treatment, rather than to a “spontaneous recovery” occurred after such a long time from the initial brain injury event. The significant neuroprotective effects obtained in our patients after intranasal NGF administration, without local or systemic side effects, open a new potential therapeutic window not only offering a rescue treatment in children with post-traumatic UWS but also in patients suffering from other neurodegenerative diseases characterized by the loss of neuronal function^77^. The ease of administration of this treatment makes it certainly worthwhile to be investigated further, mainly in the early stages from severe TBI and in patients with better baseline neurological conditions, in order to explore more thoroughly the benefits of this new therapeutic approach on cerebral function recovery. In conclusion, the development of NGF-based treatment for TBI patients is supported by the following evidences: NGF shows multiple pharmacological mechanism on target cells involved in the pathogenesis of early and chronic events caused by TBI; preclinical and clinical studies suggest a favorable safety and efficacy profile in patients with TBI; the intranasal drug delivery ensures a local pharmacological action, low invasiveness and a favorable safety profile.

Therefore, we considered this study a first step towards the development of a larger clinical project aimed at evaluating the potential effectiveness of intranasal NGF administration for improving neurological outcome and clinical functions in children with post-traumatic UWS. Although further controlled, randomized, double-blind studies are needed for a better understanding of the neuroprotective mechanisms of this neurotrophin, intranasal NGF administration appears to be a promising and safe rescuing strategy for the treatment of children with neurological sequelae due to severe TBI.

### Limitations of the study

Because of the low sample size of patients treated our data may not be generalizable to all children with post-traumatic UWS so, to minimize the selection bias of patients, clear guidelines for the assessment and treatment of this kind of patients must be used with obvious repercussions on the outcome of these children. Moreover, due to the lack of wider randomized clinical trials, more data on the efficacy, safety and possible side effects (such as pain) of intranasal hr-NGF administration are required to assess the impact of this new approach on neurocognitive development and motor function recovery in these patients. Furthermore, understanding the right dose of hr-NGF to utilize and also the administration schedule represents, even today, an important challenge to be faced. The difficulty and the limitations in treating this kind of patients may limit the development of this type of study, but defining the relationships between hr-NGF intranasal administration in the injured brain could allow the individualization of new strategies for the treatment of patients suffering from post-traumatic UWS.

### Supplementary Information

**Additional file 1. eFigure 1**: Modified Ashworth Scale.

**Additional file 2. eFigure 2**: Gross Motor Function Measure (GMFM).

**Additional file 3. eFigure 3**: Gross Motor Function Measure (GMFM) score.

**Additional file 4. eFigure 4**: Disability Rating Scale (DRS).

**Additional file 5**. Supplementary Materials: descriptions of different neuroradiological and neurological techniques used to evaluate the clinical conditions of the patients.

## Data Availability

Not Applicable.

## Abbreviations

Aβ42: Amyloid β42
BBB: Blood–brain barrier
DRS: Disability rating scale
hr-NGF: Human-recombinant NGF
EEG: Electroencephalography
GCS: Glasgow coma scale
GMFM: Gross motor function measure
GMFCS: Gross motor function classification system
IL-1β: Interleukin 1β
MRI: Magnetic resonance imaging
MAD: Mucosal atomization device
NGF: Nerve growth factor
PSD: Power spectral density
PET: Positron emission tomography scan
PICU: Pediatric intensive care unit
SPECT: Single photon emission computed tomography
SSEP: Somatosensory evoked potentials
TBI: Traumatic brain injury
TrkA: Tyrosine receptor kinase A
tDCS: Transcranial direct current stimulation
UWS: Unresponsive wakefulness syndrome
