# Supplementary figures and images for: Intranasal human-recombinant NGF administration improves outcome in children with post-traumatic unresponsive wakefulness syndrome
Source: Biol Direct. 2023 Oct 3;18:61. doi: 10.1186/s13062-023-00418-1 (PMC10546699; doi:10.1186/s13062-023-00418-1)

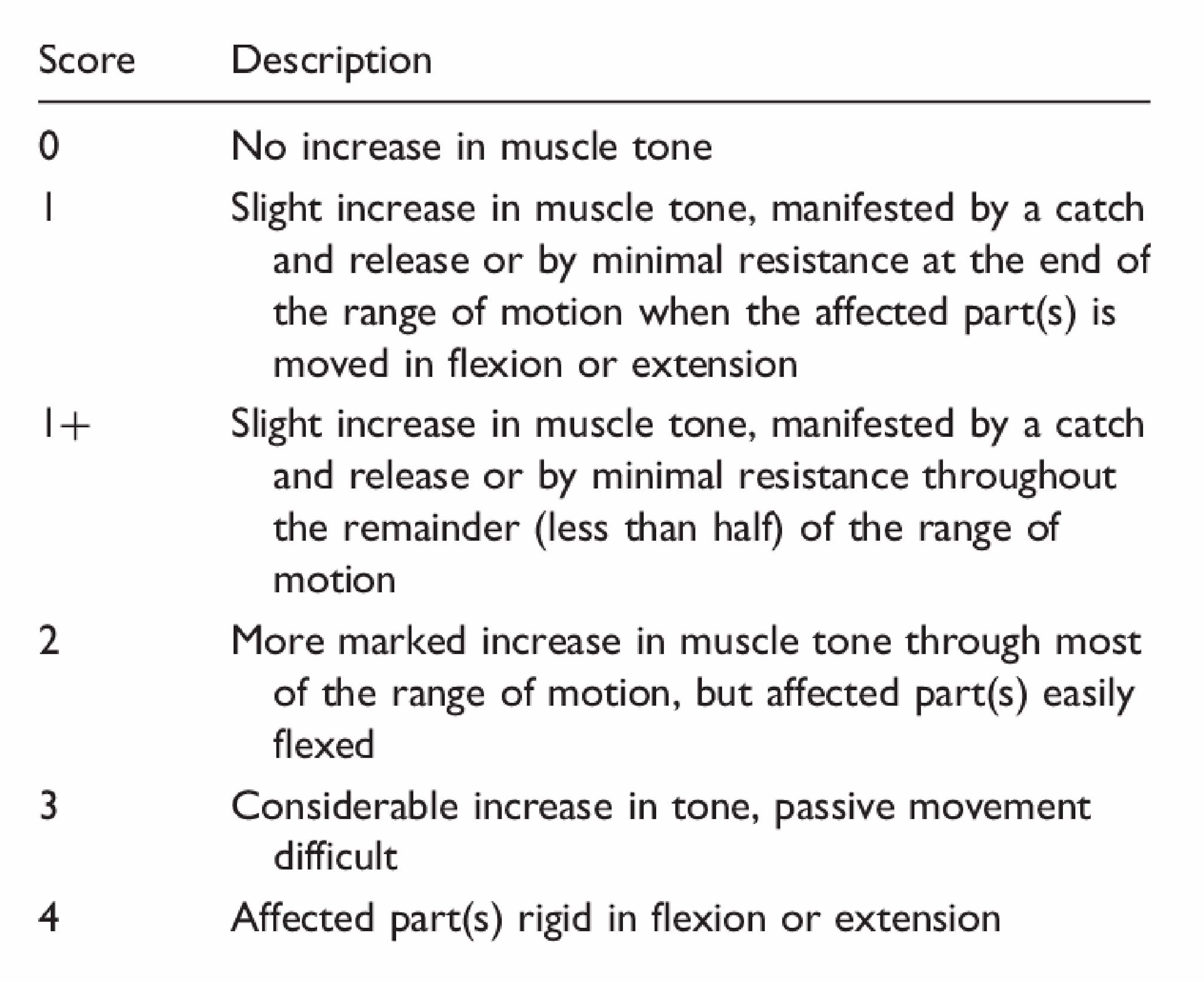

Supplement: Supplementary file 1 — Additional file 1. eFigure 1: Modified Ashworth Scale. [file 13062_2023_418_MOESM1_ESM.jpg]

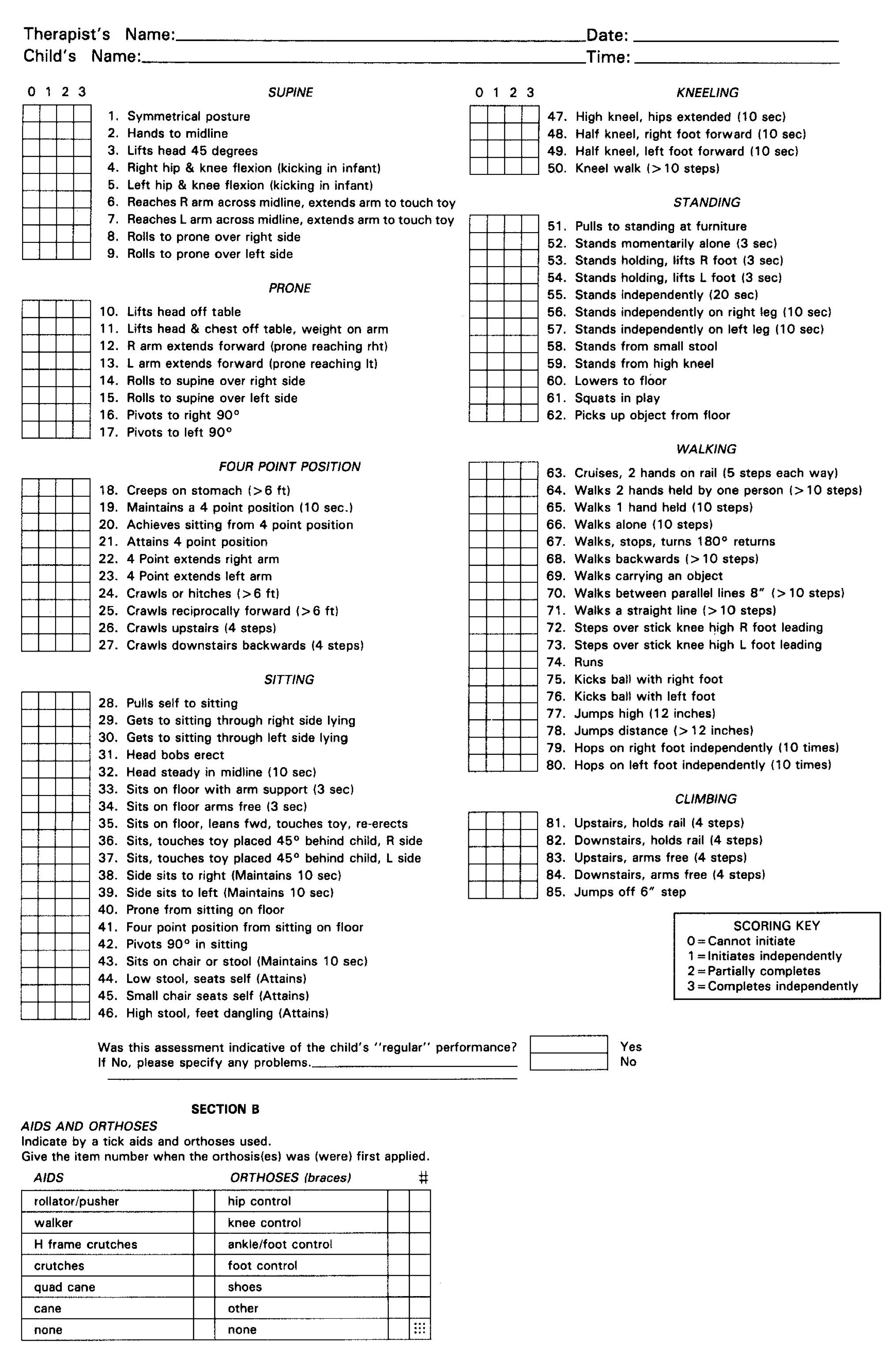

Supplement: Supplementary file 2 — Additional file 2. eFigure 2: Gross Motor Function Measure (GMFM). [file 13062_2023_418_MOESM2_ESM.jpg]

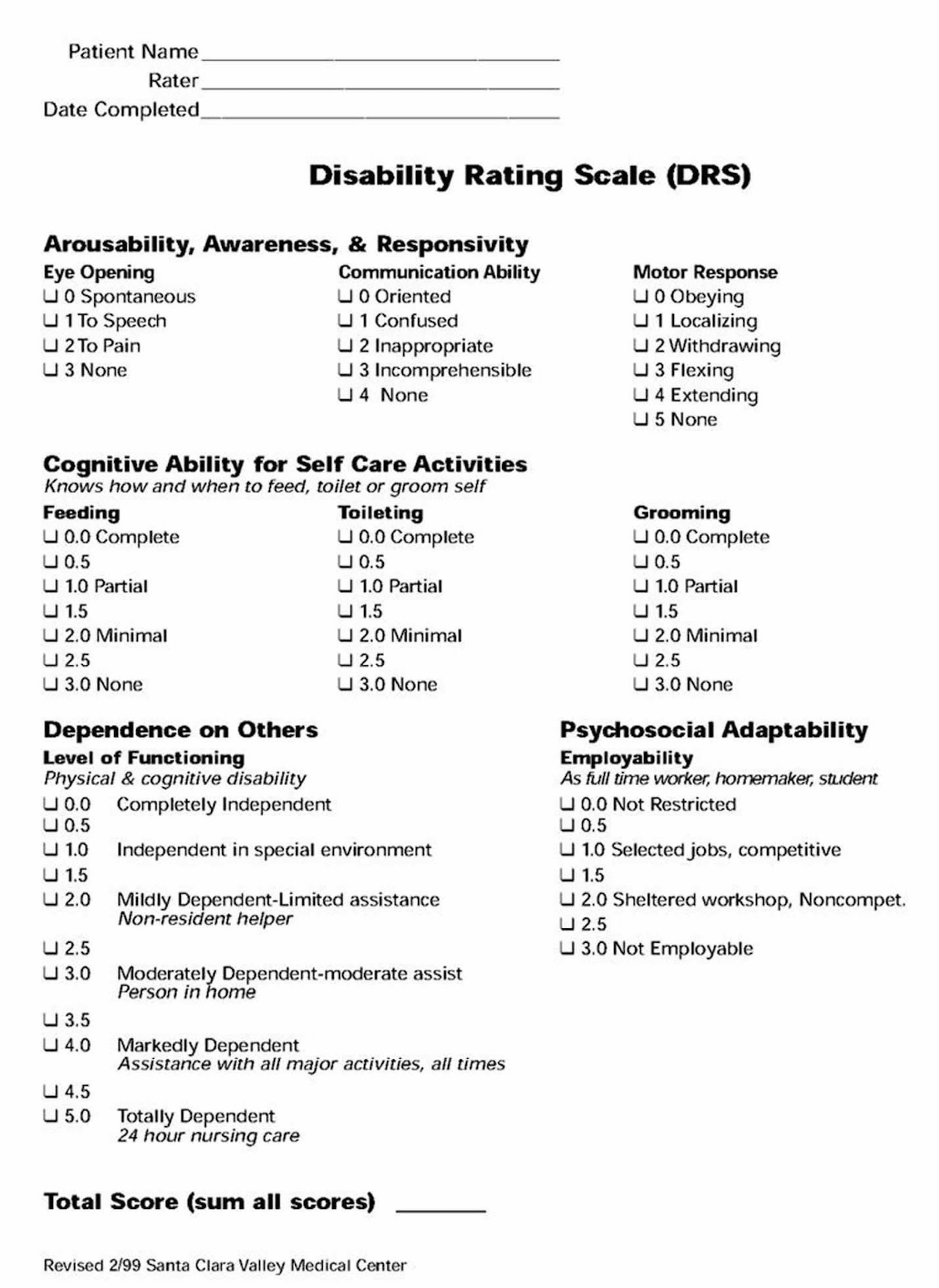

Supplement: Supplementary file 4 — Additional file 4. eFigure 4: Disability Rating Scale (DRS). [file 13062_2023_418_MOESM4_ESM.jpg]
